# Tissue Bioprinting: Promise and Challenges

**DOI:** 10.3390/bioengineering10121400

**Published:** 2023-12-07

**Authors:** Kun Liang

**Affiliations:** A*STAR Skin Research Labs (A*SRL), Agency for Science, Technology and Research (A*STAR), Singapore S138648, Singapore; kun_liang@asrl.a-star.edu.sg

## 1. Introduction

In recent years, we have witnessed remarkable progress in the field of regenerative medicine, in large part fuelled by developments in advanced biofabrication technologies such as three-dimensional (3D) bioprinting [1,2]. Bioprinting is a manufacturing approach that enables precise and controlled spatial deposition of biomaterials or “bioinks” and cells in the 3D environment based on a digitally created design. In doing so, it has the capability of generating highly complex and defined structures unparalleled by traditional engineering approaches. As such, bioprinting has the potential to address some of the most relevant clinical needs today—repair and replacement of impaired or dysfunctional tissues. In this editorial, we will explore the promises of bioprinting and consider some of the challenges that must be addressed as technology continues to evolve.

Since the initial demonstrations of material and cell deposition and patterning based on commercial inkjet printers two decades ago [3], bioprinting has advanced significantly with the development of multiple new printing approaches. The current range of bioprinting techniques includes inkjet, extrusion, stereolithography, laser-assisted, electrospinning and, more recently, free-form reversible embedding of suspended hydrogels (FRESH) bioprinting; each of them has been crafted to address specific limitations of the printing process, e.g., in terms of bioink selections, build size, speed, construct stability, print resolution, cost, etc. [4].

## 2. Promises of Tissue Bioprinting

Bioprinting holds great promise for clinical translation. One of the major benefits is its potential to enable personalized treatment. Currently, tissue transplantation is limited by the availability of suitable donors and the risk of rejection. Bioprinting offers a potential solution by allowing doctors to create customizable tissues using the patient’s own cells or induced pluripotent stem cells (iPSCs), eliminating the need for donor cells/tissues and reducing the risk of allogeneic rejection [5,6,7,8]. For tissue repair applications, such as wound healing, in situ bioprinting can also be implemented, where the printing operation is performed directly onto the defect site. This enables the accurate orientation of a construct with an architecture and topology tailored to the patient’s anatomical requirements at the site of injury [9,10,11].

Apart from medical applications, bioprinting also has the potential to advance our understanding of biological processes and functions. By fabricating biomimetic 3D tissue models that resemble native tissues, researchers can better study the interactions between cells and the extracellular microenvironment and the roles of biomolecules that are implicated [7,12,13]. Some of the more notable bioprinted models include multiple-cell-type co-cultures, organoids, and organ-on-chip systems, which provide more robust, accurate, and cost-effective platforms for toxicological investigations [14,15,16]. Similarly, 3D models of diseased tissues can also be generated via bioprinting to screen for potential drug candidates and therapeutic interventions [17,18,19] while eliminating the ethical concerns and inaccuracies associated with testing on animals.

## 3. Challenges

Despite the benefits of tissue bioprinting, there are still significant hurdles to overcome before widespread adoption. Some of these challenges include engineering tissue complexity, post-print tissue maturation and maintenance, standardized and scalable manufacture, and, for translation, a defined regulatory framework for bioprinted constructs. While geometrically complex on a macroscale, most bioprinted tissues still lack some functional elements, such as vasculature, nervous system, lymphatics, and multiple supporting cell types [2,13]. This has led to significant research efforts focusing on strategies to incorporate microstructures, such as constructing interconnected networks of channels to mimic blood vessels and promote vascularization [20,21]. Nevertheless, some key issues still need to be resolved, including vessel architecture, patency, cell distribution, and the amenability to perfusion for supporting long-term tissue viability [22,23]. From the viewpoint of bioink development, while new formulations based on novel modifications and/or combinations of existing biomaterials are created [24,25], it is also important to build a standardized bioink library coupled with quality control systems to ensure reproducibility, safety and efficacy for specific applications. Concurrently, improving bioprinting techniques that are compatible with the bioinks will be necessary to enable rapid, precise and scalable tissue fabrication. Lastly, to achieve clinical translation of tissue bioprinting, a clear and defined regulatory pathway has to be established. While it is a slow and iterative process, some key considerations can be drawn from the U.S. Food and Drug Administration (FDA) guidelines for 3D-printed medical devices [26].

## 4. Conclusions

In conclusion, the bioprinting of tissues is a key enabler for regenerative medicine in terms of the fabrication of biomimetic, personalized constructs, in vitro modelling (for both healthy and diseased) and expanding our knowledge of tissue biology. While there are still significant challenges to overcome, the outlook of bioprinting remains highly promising, and we can expect many exciting advances to emerge in the near future.
